# Barriers to the effective treatment and prevention of malaria in Africa: A systematic review of qualitative studies

**DOI:** 10.1186/1472-698X-9-26

**Published:** 2009-10-25

**Authors:** David M Maslove, Anisa Mnyusiwalla, Edward J Mills, Jessie McGowan, Amir Attaran, Kumanan Wilson

**Affiliations:** 1 Department of Medicine, University of Toronto, Toronto, Ontario, Canada; 2 Faculty of Health Sciences, Simon Fraser University, Vancouver, Canada; 3 Faculty of Health Sciences, University of Ottawa, Ontario, Canada; 4 Institute for Population Health, University of Ottawa, Ontario, Canada

## Abstract

**Background:**

In Africa, an estimated 300-500 million cases of malaria occur each year resulting in approximately 1 million deaths. More than 90% of these are in children under 5 years of age. To identify commonly held beliefs about malaria that might present barriers to its successful treatment and prevention, we conducted a systematic review of qualitative studies examining beliefs and practices concerning malaria in sub-Saharan African countries.

**Methods:**

We searched Medline and Scopus (1966-2009) and identified 39 studies that employed qualitative methods (focus groups and interviews) to examine the knowledge, attitudes, and practices of people living in African countries where malaria is endemic. Data were extracted relating to study characteristics, and themes pertaining to barriers to malaria treatment and prevention.

**Results:**

The majority of studies were conducted in rural areas, and focused mostly or entirely on children. Major barriers to prevention reported included a lack of understanding of the cause and transmission of malaria (29/39), the belief that malaria cannot be prevented (7/39), and the use of ineffective prevention measures (12/39). Thirty-seven of 39 articles identified barriers to malaria treatment, including concerns about the safety and efficacy of conventional medicines (15/39), logistical obstacles, and reliance on traditional remedies. Specific barriers to the treatment of childhood malaria identified included the belief that a child with convulsions could die if given an injection or taken to hospital (10/39).

**Conclusion:**

These findings suggest that large-scale malaria prevention and treatment programs must account for the social and cultural contexts in which they are deployed. Further quantitative research should be undertaken to more precisely measure the impact of the themes uncovered by this exploratory analysis.

## Background

Each year, malaria accounts for up to 1 million deaths worldwide, mostly in children under five [1]. In 2002, there were as many as 500 million episodes of clinical *Plasmodium falciparum *malaria infection, and more than two thirds of these cases were in Africa [2]. More recently, malaria related morbidity and mortality have been significantly worsened by the emergence of widespread drug-resistance [3].

During the past decade, numerous large-scale initiatives have been undertaken with the goal of reducing or eradicating the burden of malaria in the developing world. These include among others the Global Fund for AIDS, Tuberculosis, and Malaria (GFATM), the Roll Back Malaria Partnership (RBM), and the Medicines for Malaria Venture (MMV). The ambitious goals set by these programs for reducing the burden of malaria in the near future appear unlikely to be met [4].

The success of antimalarial interventions requires appropriate coordination of efforts, as well as acceptance at the community and individual levels. Numerous barriers to adequate malaria control programs now exist, including the increasing prevalence of drug and insecticide resistance, the high rate of HIV co-infection, climate change, and civil unrest [5]. Added to these are the potential barriers posed by the local cultural contexts in which those at risk of malaria live. Previous experience from the field of HIV/AIDS has shown that local perceptions of this disease and its causative agent are strongly influenced by cultural beliefs, and that these perceptions must be considered in the development of prevention and treatment programs [6,7]. Cultural beliefs are likely to similarly influence the treatment and prevention of malaria in Africa. Accounting for these should enhance the efficacy and scope of malaria control programs. To our knowledge, no study has systematically compiled the results of qualitative research on cultural beliefs about malaria in sub-Saharan Africa, across a range of age groups and countries.

Qualitative studies provide greater insights into personal experiences than quantitative methods [8]. An excellent review of qualitative studies done between 1996 and 2000 explored qualitative data "pertaining to the home management of illness episodes of malaria in sub-Saharan Africa" [9]. This study updates the results of that study, uses formal systematic review methods for qualitative studies, and does not restrict the analysis to home management of episodes.

## Methods

We conducted a systematic review of qualitative studies using methods previously described [10]. We identified articles from a literature search, and systematically extracted relevant themes identified in each article. These themes formed the basis of a checklist used to determine the frequency with which each theme had been identified in each of the papers.

### Search Strategy

To identify articles for inclusion in our analysis, we searched Medline (1966 to June, 2009) and Scopus (1968 to June, 2009). The complete search strategy is outlined in the Appendix. Reports produced by government, non-governmental organizations, and industry that were not subject to peer review, so-called "grey literature", were not included because the methodological quality of such accounts is difficult to ascertain.

### Selection of abstracts

Two of us (AM, DM) independently screened a representative sample of 250 articles, in order to develop inclusion and exclusion criteria. The primary author then screened the remaining articles, using the criteria developed. We retrieved all the articles describing studies that made any use of qualitative methods (semi-structured, structured or unstructured interviews, or focus group discussions) to investigate the knowledge, beliefs, attitudes, and practices related to malaria amongst residents of an area where malaria is prevalent. Where no abstract was available, we retrieved articles if they included the term "malaria" in the title, with at least one of "beliefs", "knowledge", "attitudes", or "practice". We added to these the papers that were identified through a review of references.

To ensure we included purely observational qualitative data representing the views of the majority of patients and caregivers, we examined in detail the methods sections of all of the articles selected from the review of abstracts. We excluded the remaining quantitative studies, the second instances of articles found in both databases, those in which an experimental or interventional design was used, and those focused on either travellers, drug sellers, adverse drug events, or malaria in pregnancy. Of the remaining articles, we selected only those written in English, describing studies conducted in sub-Saharan African countries.

### Extraction of themes

Two of us (AM, DM) independently reviewed the results sections of a representative sample of the articles selected for analysis, and extracted themes relating to beliefs about the causes, treatment, and prevention of malaria. A separate author (KW) categorized the extracted themes to produce a scoring checklist. With this checklist, all of the included articles were reviewed, to determine which articles identified each of the themes. Discrepancies were resolved by consensus.

For studies carried out amongst a population where there is no direct translation of the term "malaria", the local terminology for febrile illness was assumed to refer to malaria. This assumption was only made in cases in which the original authors indicated that this was warranted, and in which they themselves had used the terms interchangeably. For articles in which both qualitative and quantitative data were reported, only the qualitative data were used in the analysis.

For a representative sample of articles, we also extracted data on study methodology and assessed quality by means of the CASP critical appraisal tool [11]. As the outcome of this appraisal did not affect our study inclusion criteria, or the weighting of their findings in our analysis, the results are not reported in detail here.

### Statistical analysis

For the initial selection of abstracts, we estimated level of agreement between reviewers using the κ statistic.

## Results

### Study selection and characteristics

**Figure 1:**
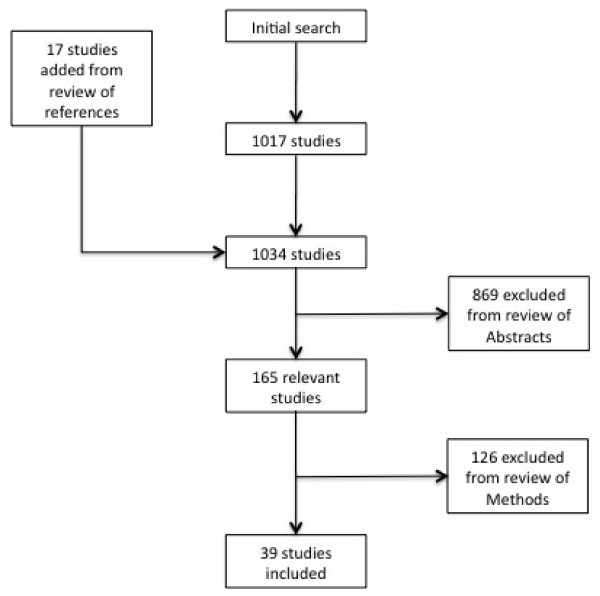
**Flow diagram of the studies used in the analysis**.

Our search identified 1017 articles (See Figure 1), and the review of references identified an additional 17 articles. Of these, 869 were excluded based on the review of abstracts. The κ statistic for the initial selection was 0.77, indicating excellent agreement. Of the remaining 165 studies, a further 126 were excluded by consensus because they used only quantitative methodologies, were conducted in non-African locales, or pertained to either travellers or pregnant women. A total of 39 articles were included in the analysis [12-50].

The characteristics of the studies selected are shown in Tables 1 and 2. The studies were done in 12 different countries, with the majority conducted in either East Africa (Kenya, Tanzania, Uganda - 18 studies), or West Africa (Benin, Burkina Faso, Côte d'Ivoire, Ghana, Nigeria - 13 studies). The others were conducted in Southern Africa (Malawi, Zambia - 5 studies), Ethiopia (2 studies), and Sudan (1 study). The articles were published between the years 1992 and 2008, with a median year of publication of 2002. Four of the 39 included articles primarily focused on adults, 19 articles primarily focused on children, and 16 articles were of mixed focus. Twenty-five studies took place amongst rural or semi-rural populations, 11 were conducted in both rural and urban settings, and 3 were limited to urban areas alone. Twenty-seven of the 39 studies included as respondents the caregivers of young children. Other groups studied included non-caregiver adults, health workers, traditional healers, community leaders, and school-aged children. Qualitative data were obtained mostly by means of focus group discussions and interviews.

**Table 1:** Characteristics of qualitative studies in children

Name	Year	Country	Setting	Population	Primary methods (n)	Disease terminology
Mwenesi	1995	Kenya	Peri-urban, urban, rural	Mothers with ill children Adults	Interviews Key informant interviews	*homa*

Mwenesi	1995	Kenya	Urban township, slum, rural	People in the community	Key informant interviews (60)	Malaria, *homa, dege*

Makemba	1996	Tanzania	Rural	Traditional healers Parents who had taken their children to healers	14 interviews 3 focus groups	*Degedege, homa ya kawaida*

Ahorlu	1997	Ghana	Rural	Caregivers of children under 10	8 focus groups (1057)	*Asra, asraku, atridi*

Williams	1999	Zambia	Rural	Mothers of children under 5 Father of children under 5 Others*	9 focus groups 7 focus groups 36 in-depth interviews	*thupi kuphya, malungo, cilunguzi*, malaria, *cinthu, cawoka*, *malaria, yakulu*, 'big malaria'

Baume	2000	Zambia	Rural and urban	Caregivers of children under 5 with recent febrile illness	Semi-structured interviews (154)	-

Muela	2002	Tanzania	Rural and urban	Mothers of children under 5	In-depth (81) and follow-up (10) interviews	Malaria, *homa, homa ya mbu, homa kali, degedege, malaria ya kichwa*

Comoro	2003	Tanzania	Urban, peri-urban, rural	Mothers of sick children under 5, Health workers	10 focus groups (85) 6 focus groups (43)	*Homa ya malaria, degedege*

Muula	2004	Malawi	Peri-urban	Primary school pupils aged 12-18	4 focus groups (40)	-

Nsungwa-Sabiiti	2004	Uganda	Rural	Mothers, fathers, grandparents	10 focus groups	*omutsutsa*

Akogun	2005	Nigeria	Rural	Mothers	6 focus groups	*zazzabi*

Kaona	2005	Zambia	Rural	Mothers, fathers, grandparents, health care providers, community members	12 focus groups (97), 29 key informant interviews	*malaria, inzekema, impepo, sinsa, ichinzekema*

Falade	2006	Nigeria	Urban and rural	Mothers, fathers, and caregivers of children under 10	Focus groups	*Iba, iba lasan*

Kamat	2006	Tanzania	Rural	Mothers/caretakers	In-depth interviews (45)	*Homa ya kawaida, homa kali, homa ya malaria, degedege*

Makundi	2006	Tanzania	Rural	Mothers/caregivers Traditional healers	2 Focus groups In-depth interviews	*Malaria ya kawaida, malaria kali, degedege*

Malik	2006	Sudan	Rural	Mothers	10 Focus groups	-

Montgomery	2006	Tanzania	Urban and rural	Parents/caretakers of children under 5 Health practitioners	Interviews (79) Interviews (55)	-

Beiersmann	2007	Burkina Faso	Rural	Mothers Guérisseurs, nurses, traditional birth attendants	17 Focus groups Semi-structured interviews (17)	*Sumaya, dusukun kono*

Deressa	2007	Ethiopia	Rural	Mothers of children under 5 Fathers of children under 5	3 Focus groups 3 Focus groups	*busa*

* Parents, drug vendors, traditional healers, and community- and facility-based health workers

**Table 2:** Characteristics of qualitative studies in adults and mixed populations

Name	Year	Country	Setting	Population	Primary methods (n)	Disease terminology
Adult focus						

Agyepong	1992	Ghana	Rural	Men and women over 20 years old	6 focus groups	*Asra, asraku*

Stevens	1995	Tanzania	Urban, peri-urban	Adults, community leaders	8 focus groups (94), 72 focused discussions (175)	malaria

Rashed	1999	Benin	Rural	Parents, community elders, non-western healers, health care providers	23 focus groups (252), 8 semi-structured interviews	*Ouevozon*

Nyamongo	2002	Kenya	Rural	Adults aged 18 to 80	Interviews (35)	-

Mixed focus						

Helitzer-Allen	1993	Malawi	Rural	Mothers, pregnant women, husbands, health workers, community leaders	160 in-depth interviews^†^ 24 focus groups^††^	*Malungo*

Agyepong	1994	Ghana	Rural and urban	Caregivers of children under 5 years	Interviews (471)	*Asra, asraku*

Kengeya-Kayondo	1994	Uganda	Rural	Women, mothers, female caregivers	5 focus groups (42), 395 semi-structured interviews, 64 key informant interviews	-

Winch	1996	Tanzania	Rural	Group meetings with local government officials, religious leaders, teachers, and health workers,	40 unstructured interviews and focus groups, pile sorting with 8 respondents	*Homa*, *homa kali*, *homa ya kuchemka*, *homa ya malaria*, *degedege*

Muela	1998	Tanzania	Semi-rural	Adult malaria patients, caretakers of children under 5, mothers, villagers, traditional healers	6 focus groups, 103 interviews	Malaria, *homa*, *homa ya malaria*, *degedege*

Munguti	1998	Kenya	Rural	Heads of households reporting a case of malaria within the previous 2 weeks	Structured interviews (463)	-

Mixed focus (cont'd)						

Oberlander	2000	Tanzania	Rural village	-	Participant observation, informal group discussion, ethnographic interviews	*Degedege, mchango, kibwengo*, malaria

Brieger	2001	Nigeria	Urban	Adults, child caregivers	36 focus groups, 154 interviews	*Iba*, malaria, fever, malaria fever

Nuwaha	2002	Uganda	Rural, partly-urban	Men, women, heads of households	4 focus groups	*omushwija, omussuja*

Okrah	2002	Burkina Faso	Rural, partly-urban	Caregivers of children under 5, adult community members	10 focus groups, 9 key informant interviews	*Soumaya*

Adongo	2005	Ghana	Rural	Women, men, couples, mothers, healers, bednet vendors	8 focus groups, 98 interviews	*Pua, feber, nienga, zumzuri*

Eriksen	2005	Tanzania	Rural and urban	Mothers, fathers, health workers	12 focus groups	-

Onwujekwe	2005	Nigeria	Rural	Men, women, youths	9 focus groups	*iba*

Deressa	2007	Ethiopia	Rural	Mothers of children under 5 Men with at least one child	3 Focus groups 4 Focus groups	*busa*

Essé	2008	Côte d'Ivoire	Rural	School children aged 10-14 Health practitioners, health facility staff, local healers, religious leaders	6 Focus groups 15 Key informant interviews	*Djèkouadjo, djékadjo, ewuego, anumą*

Idowu	2008	Nigeria	Rural	Adults	Focus groups	*Iba otutu*

^†^interview target groups -- pregnant women, women who had recently given birth, chiefs and village headmen, husbands of pregnant women, traditional birth attendants, health worker, traditional advisors, and traditional healers

^††^focus group target groups -- pregnant women, women who had recently given birth, and husbands of pregnant women

### Local terminology for febrile illnesses

In many of the studies included in the analysis, the local vernacular of disease terms was used. While these languages often lacked a direct translation for the English word "malaria", they included a single-word term or phrase of similar meaning. Examples of this include the terms *asra *(Ghana), *homa *(Tanzania and Kenya), *soumaya *(Burkina Faso), and *omusujja *(Uganda). While these terms translate roughly to the English word "fever", their meanings encompass a number of other symptoms, such that they correspond closely to the clinical presentation of malaria [13,31].

### Barriers to Prevention

The themes extracted from the articles are shown in Tables 3 and 4 [see additional file 1 and 2]. Twenty-nine of the 39 articles included identified potential barriers to effective malaria prevention. Numerous barriers to the use of bednets were identified, including cost, and ease of use. As a respondent from a study in Ghana explained, "The problem associated with a bednet is that you sweat a lot when sleeping under it. I don't know the price now but I think only a few people can afford it" [15]. Concerns about repellents reported in one article included unpleasant side effects from coil smoke and insecticide sprays [48].

Twelve articles reported the use of ineffective prevention practices, such as eating a balanced diet, drinking herbal teas, wearing charms or amulets, and vaccinating children. Seven articles identified the belief that malaria cannot be prevented.

Twenty-seven out of the 39 articles analyzed described the belief that malaria is caused by factors other than mosquitoes and *Plasmodium *parasites (median publication year 2002). Of these, the most frequently identified included environmental factors (excessive heat, wind, or cold), dietary factors (eating oily foods, certain fruits and grains, or too much of the same foods), drinking or bathing in dirty water, and supernatural causes (witchcraft, sorcery, and possession by spirits).

In 6 articles, mosquitoes were not mentioned amongst the causes of malaria reported by the study participants. These beliefs were shown in some studies to have an important impact on prevention:

*Malaria cannot be prevented because we were all born with it and it grows in us once a while, it manifests itself by giving us fever. When we treat it, we get better but the malaria is still there and will come again but to prevent it from coming you have to take an enema every day *[15].

A related theme that was identified in 19 of the articles was the tendency to ascribe syndromes that likely represent severe or complicated malaria, including convulsions and anemia, to non-malarial causes (median publication year 2002). Specifically, convulsions were frequently ascribed to witchcraft, possession by spirits, or exposure to birds and animals with supernatural properties. This theme was identified most frequently in articles focused on malaria in children.

### Barriers to Treatment

Thirty-seven of the 39 articles identified barriers to active treatment. The most frequently identified concern about conventional therapy, reported in 10 of the articles analyzed, was the belief that a child with convulsions could die if given an injection. Three of these articles also identified the belief that a sick child could die if taken to hospital. Yet another article reported the belief that antimalarials should be withheld from a child with fits. Nearly all of the studies that identified these themes were conducted in East Africa (median publication year 2000).

Other reported barriers to treatment included beliefs about the efficacy and use of conventional medicines, and beliefs about the role of traditional therapies. A total of 17 articles described a preference for traditional remedies in cases where the illness was believed to be caused by spirits or witchcraft, when complications occurred, or as a means of removing the root cause prior to supportive care in hospital. This was particularly evident in the case of childhood convulsions, which were often believed to be due to spirits or witchcraft, and were accordingly treated by traditional means:

*"Degedege is caused by bad spirits. In this realization, spirits that cause convulsion must be removed first so that Western and other medication can work in treating the child. That is why we start at the traditional healer for treatment of a convulsed child and later we take him or her to hospital" *[32].

Failure of hospital treatment was identified in 2 studies as an indication that the illness was due to witchcraft. Three studies reported beliefs that the taste and colour of pills was a reflection of their efficacy, specifically that bitter medications such as chloroquine were more effective.

We identified numerous behavioural and pragmatic barriers to the use of conventional medicines. Barriers reported about use of treatment facilities included distance from the facility, perceived quality of treatment provided, and fear of scolding from clinic staff:

*During the time the child was sick, I was also sick. The other thing is that it is very far to the health center. Unless I had a bicycle, I could not have taken her there*.

*...And those people at the clinic never really examine our children. They just write what we tell them. If you ask questions, they just shout at you*... [17].

The prohibitive cost of treatment was described in 12 articles. Six articles identified the practice of stopping medications once symptoms had ceased, often as a means of rationing pills to preserve a supply for future illness episodes:

*...sometimes just after administering two times you find the child recovers, starts playing. So you stop and keep the medicine, in case the child falls ill again *[17].

A total of 23 articles described conventional medicines and health care clinics as second line treatment options. Nearly all of the articles described the use of traditional healing methods, including consultation with traditional healers, use of herbal remedies, sponging and bathing, and various forms of fumigation. These methods were often described as first line treatment for malaria, before conventional treatments are employed:

*Usually patients are first given herbs especially mululuza *(a popular herb used for omusujja) *then they go on to tablets from shops and eventually they take the patients to the hospital, especially if the omusujja fails to respond to the herbs/tablets *[30].

## Discussion

We systematically reviewed qualitative studies examining the local understanding of malaria in sub-Saharan African countries, as a means of identifying important beliefs and practices that might pose barriers to effective malaria treatment and prevention. To our knowledge, ours is the first systematic review of qualitative studies in this area. While qualitative methods remain useful in exploratory research and hypothesis generation, their results when taken in isolation are of limited value in making more general claims. Our method addresses this shortcoming by using a content analysis approach to combine the results of many qualitative studies conducted in different settings amongst various populations [10].

Our results have importance for public health planners and local physicians. We found that cultural beliefs regarding the cause of malaria may differ from scientific explanations, and that first-line treatments are frequently based on traditional practices. These findings indicate that public health prevention and treatment campaigns need to include culturally sensitive strategies that provide education in the context of local understanding and beliefs.

The results of our analysis show that while the malaria-mosquito link is recognized in malaria-endemic regions of sub-Saharan Africa, a number of alternate causative mechanisms are also endorsed. Quantitative data from the studies included in our analysis support this finding, showing that up to 80% of survey respondents cite causes of malaria that do not implicate transmission by mosquitoes [12-14,37,45,50]. Similarly, only 8-27% of respondents related common complications of malaria such as convulsions and anemia, to mosquitoes or malaria [12,40]. This finding has important implications for malaria control programs, which rely heavily on vector control strategies, including the use of insecticide-treated bednets (ITNs). Users must believe in a mosquito-based etiology of malaria to be sufficiently motivated to purchase or otherwise acquire ITNs, and adhere to their use.

Improved understanding of malaria transmission is also likely to result in better adherence to effective prevention strategies. For one example, a study of post-partum women in Tanzania showed that a high level of knowledge of malaria transmission was positively correlated with the use of ITNs [51]. Taken together, these findings along with those from our study, support a role for education as a key component of any project aiming to reduce the burden of malaria in endemic areas in Africa.

We also identified a number of unproven prevention practices that are potential consequences of the beliefs about causation. Quantitative data from one of the studies included in our analysis showed that while 19% of mothers believed charms or amulets could prevent convulsions in their children, only 9% subscribed to mosquito avoidance for this purpose [40]. While most of these practices are likely not to be harmful, they pose a possible threat to successful malaria prevention by supplanting the use of more effective measures. Use of such methods may also foster a false sense of protection amongst those who apply them.

The WHO malaria treatment guidelines currently recommend artemisinin-based combination therapies (ACTs) as first-line treatment for both uncomplicated and severe malaria [52]. Access to ACTs, however, is still limited for many populations, amongst which traditional methods remain the primary source of care. The results of this review indicate that traditional remedies are often used in the treatment of both uncomplicated and severe malaria. Some of these methods, such as tepid sponging for children with high fevers, have demonstrated some benefit in a small number of clinical trials [53]. For the most part, however, their efficacy remains unexamined, and thus uncertain. While certain herbal treatments may be of benefit - artemesinin itself is a derivative of the Chinese plant *Artemisia annua *- many traditional therapies may be ineffective, or even harmful. Their use as first-line treatment should be countered with the implementation of available and effective antimalarials.

Our analysis also shows that traditional healers are frequently consulted in the treatment of malaria, likely as a consequence of their role within local cultures, and of geographic and financial barriers to accessing treatment from health clinics. Survey data from one study in our analysis indicated that 70% of mothers sought treatment for febrile children from traditional healers [17]. As such, they are particularly well positioned to affect changes in the treatment and prevention practices of their patients. Local healers should be closely involved in malaria treatment programs, and should be encouraged to incorporate the use of evidence-based antimalarials into their practice. The reach and influence of traditional healers as primary and first-line caregivers should be utilized in order to improve timely and open access to ACTs.

The results of this review describe significant scepticism around the use of conventional medicines in the treatment of malaria, and in particular in the treatment of severe malaria in children. We identified the concern amongst caregivers of young children that giving an injection to a child with convulsions can be fatal. This belief may in fact be a consequence of local treatment practices, in which seeking hospital care is delayed in deference to traditional treatments. Death following an injection may therefore be a result of the high mortality of late-presenting severe malaria, rather than of the injection itself. Current guidelines continue to support the use of parenteral antimalarials in children with severe malaria, as well as the use of rectal or parenteral benzodiazepines for convulsions whenever this is feasible [52].

We found 6 articles that described the practice of discontinuing malaria treatment once symptoms were seen to resolve, often as a means of rationing medicines for future illness episodes. This finding is particularly worrisome, as poor adherence to treatment is a risk factor for treatment failure [3], and could promote the emergence of drug resistant parasites.

Our study has several strengths and limitations to consider in the interpretation of its findings. We used established systematic review techniques to identify and extract data in an unbiased manner. Using a methodology published elsewhere, we coded and extracted themes, resolving disagreements through 3^rd ^party arbitration or consensus [10]. However, our analysis relied on the assumption that the local terminology for febrile illnesses used in many of the articles referred specifically to malaria. It is possible that these terms in fact describe febrile illnesses other than malaria. In the case of the term *degedege*, which was assumed to refer to severe malaria or cerebral malaria in children, it is possible that non-malarial causes of febrile convulsions in children (such as meningitis, encephalitis, or high fever alone from any cause) were inadvertently included in our analysis. Our justification for using local terminology in our analysis is two-fold. First, the substitution was carried forward only for those studies in which the original authors, with significant exposure to the local language and culture under investigation, also made the substitution. Second, given that the studies included were conducted in malaria-endemic stable high-transmission areas, it is likely that malaria accounts for more episodes of fever in these regions than any other cause. Indeed, the current WHO treatment guidelines for malaria recommend empirical antimalarial treatment for children under five with a temperature of >37.5°C, as malaria is the most frequent cause of fever in this age group [52].

Other potential limitations are related to the studies included in our analysis. First, it is possible that the search strategy used did not adequately capture the most appropriate studies for inclusion, especially since many of these may have been published in smaller journals and may not have been included in the indexes we searched. Our review of key references was used to mitigate against this possible bias. Second, there were limited data in the included studies by which to assess the rigor of the experimental methods used. The extracted data may therefore be of heterogeneous quality. Nonetheless, consistency of themes was observed amongst the various studies included. Moreover, amongst those studies that did include quantitative data (23/39), these tended to support the findings of the qualitative components. Third, the countries in which the included studies were carried out are themselves a heterogeneous group, with significant cultural and linguistic differences between them. Though consistency of themes from studies in different countries was found, our findings should be interpreted and applied on a case-by-case basis according to the cultural context of a particular region. Finally, while it is likely that the themes identified in this study are important amongst the populations included, little can be gleaned about beliefs that were not reported. Thus, our method is specific, but not sensitive, for the identification of beliefs about malaria amongst the populations studied. Furthermore, the degree to which these findings can be applied more broadly to other populations remains unknown.

## Conclusion

We systematically identified barriers to effective malaria treatment and prevention that are consequences of logistical impediments, scepticism of conventional treatments, preference for traditional modalities, and incomplete understanding of malaria causation and transmission. Our findings from these qualitative studies should be used to formulate questionnaires and other research tools for use in larger quantitative studies, in order to better determine the impact of these important cultural perceptions on the prevention and treatment of malaria in sub-Saharan Africa. Accounting for the cultural beliefs and practices of sub-Saharan populations at risk of malaria is likely to enhance the effectiveness of large-scale aid programs, and ensure that the financial and logistical resources being committed to combat malaria are being appropriately allotted.

## Competing interests

The authors declare that they have no competing interests.

## Authors' contributions

DM contributed to the conception and design of the study, acquisition, analysis, and interpretation of data, and drafting and revision of the manuscript. AM contributed to the conception of the study, and the acquisition and analysis of data. EJM contributed to the conception and design of the study, interpretation of data, and revision of the manuscript. JM contributed to the acquisition of the data. AA contributed to the conception of the study and interpretation of data. KW contributed to the conception and design of the study, analysis and interpretation of data, and revisions of the manuscript. All authors read and approved the final manuscript.

## Appendix

### Search Strategy

We searched both Medline and Scopus from 1996 to May, 2009, using MeSH terms and textwords for "malaria", and combined these with MeSH and textwords terms for "National Health Programs", "communicable disease control" and "referral and consultation." This set of results was further focused by combining its results with MeSH terms for "health knowledge, attitudes, Practice" and "cultural characteristics" and textwords for "attitudes" and "beliefs". To mitigate any potential publication bias, we also reviewed the references of key papers.

## Pre-publication history

The pre-publication history for this paper can be accessed here:

http://www.biomedcentral.com/1472-698X/9/26/prepub

## Supplementary Material

Additional file 1 **Themes identified from articles focused on children**. Table shows the themes extracted from articles focused on children. Click here for file

Additional file 2 **Themes identified from articles focused on adults, and with mixed focus**. Table shows the themes extracted from articles focused on adults, and with mixed focus. Click here for file
