# Supplementary material for: Barriers to the effective treatment and prevention of malaria in Africa: A systematic review of qualitative studies
Source: BMC Int Health Hum Rights. 2009 Oct 25;9:26. doi: 10.1186/1472-698X-9-26 (PMC2782321; doi:10.1186/1472-698X-9-26)
Supplement: Additional file 1 — Themes identified from articles focused on children. Table shows the themes extracted from articles focused on children. [file 1472-698X-9-26-S1.pdf]

Additional file 1. Themes identified from articles focused on children

	Kenya	Kenya	Tanzania	Ghana	Zambia	Zambia	Tanzania	Tanzania	Malawi	Uganda	Nigeria	Zambia	Nigeria	Tanzania	Tanzania	Sudan	Tanzania	Burkina Faso	Ethiopia
	Mwenesi 1995	Mwenesi 1995 (2)	Makemba 1996	Ahorlu 1997	Williams 1999	Baume 2000	Muela 2002	Comoro 2003	Muula 2004	Nsungwa-Sabiiti 2004	Akogun 2005	Kaona 2005	Falade 2006	Kamat 2006	Makundi 2006	Malik 2006	Montgomery 2006	Beiersmann 2007	Deressa 2007
**Barriers to prevention of malaria**																			
Belief that malaria cannot be prevented	✓	✓		✓															
Belief in non-mosquito etiology		✓	✓	✓			✓		✓		✓		✓	✓	✓			✓	
Ineffective prevention measures used			✓	✓			✓								✓			✓	
Logistical barriers (cost, availability)				✓															
**Barriers to active treatment of malaria**																			
Beliefs about conventional treatments that may cause harm																			
Injections are dangerous if applied to a weak body							✓												
A child with convulsions could die if she/he gets an injection		✓	✓			✓		✓							✓			✓	
Antimalarials and/or antipyretics should be withheld from children with fits	✓																		
Taking a sick child to hospital could result in death		✓						✓											
Beliefs that may limit access to conventional treatment																			
Colour and taste of pills reflect efficacy					✓														
Medications must agree with ones blood/strength							✓												
Malaria due to spirits or witchcraft must be treated by a traditional healer						✓													
Failure of hospital treatment indicates the disease is due to witchcraft							✓												
Limited understanding of natural history and complications*		✓	✓	✓		✓	✓	✓		✓	✓	✓		✓	✓			✓	

Complications* of malaria should be treated with traditional remedies	✓	✓	✓			✓		✓		✓	✓		✓		✓				
Fear of adverse events including death from use of SP†																			
Behaviours that may reduce timely access to conventional treatment																			
Western medicines used as second resort										✓		✓							
Health care facility used as second resort		✓		✓		✓		✓		✓	✓	✓	✓	✓	✓	✓		✓	
Treatment for febrile convulsions not sought for 1-3 days			✓																
Medications stopped when symptoms cease						✓		✓					✓						
Hospital treatment sought after cause is relieved by traditional methods			✓				✓	✓							✓				
Treatment plan must be approved by fathers/husbands				✓						✓							✓		
Conventional treatment failure leads to switch to herbals/traditional treatment											✓								
Use of traditional/herbal remedies	✓	✓		✓	✓	✓		✓		✓	✓	✓	✓		✓			✓	✓
Pragmatic obstacles to accessing conventional treatment																			
Distance from facility/practitioner						✓										✓			
Costs	✓					✓										✓	✓	✓	

*convulsions, anemia, splenomegaly
†Sulphadoxine-pyrimethamine
