# Supplementary material for: Barriers to the effective treatment and prevention of malaria in Africa: A systematic review of qualitative studies
Source: BMC Int Health Hum Rights. 2009 Oct 25;9:26. doi: 10.1186/1472-698X-9-26 (PMC2782321; doi:10.1186/1472-698X-9-26)
Supplement: Additional file 2 — Themes identified from articles focused on adults, and with mixed focus. Table shows the themes extracted from articles focused on adults, and with mixed focus. [file 1472-698X-9-26-S2.pdf]

Additional file 2. Themes identified from articles focused on adults, and with mixed focus

	Primary focus on adults				Mixed focus															
	Ghana	Tanzania	Benin	Kenya	Malawi	Ghana	Uganda	Tanzania	Tanzania	Kenya	Tanzania	Nigeria	Uganda	Burkina Faso	Ghana	Tanzania	Nigeria	Ethiopia	Côte d'Ivoire	Nigeria
	Agyepong 1992	Stevens 1995	Rashed 1999	Nyamongo 2002	Helitzer-Allen 1993	Agyepong 1994	Kengeya-Kayondo 1994	Winch 1996	Muela 1998	Munguti 1998	Oberlander 2000	Brieger 2001	Nuwaha 2002	Okrah 2002	Adongo 2005	Eriksen 2005	Onwujekwe 2005	Deressa 2007	Essé 2008	Idowu 2008
**Barriers to prevention of malaria**																				
Belief that malaria cannot be prevented	✓				✓										✓				✓	
Belief in non-mosquito etiology	✓		✓		✓	✓	✓	✓	✓	✓	✓	✓	✓	✓	✓		✓	✓	✓	✓
Ineffective prevention measures used			✓		✓		✓		✓					✓	✓				✓	
Logistical barriers (cost, availability)		✓	✓		✓								✓	✓				✓		
**Barriers to active treatment of malaria**																				
Beliefs about conventional treatments that may cause harm																				
Injections are dangerous if applied to a weak body																				
A child with convulsions could die if she/he gets an injection								✓	✓		✓		✓							
Antimalarials and/or antipyretics should be withheld from children with fits																				
Taking a sick child to hospital could result in death								✓												
Beliefs that may limit access to conventional treatment																				
Colour and taste of pills reflect efficacy					✓				✓											
Medications must agree with ones blood/strength	✓				✓															
Malaria due to spirits or witchcraft must be treated by a traditional healer					✓			✓	✓						✓					
Failure of hospital treatment indicates the disease									✓											

is due to witchcraft																				
Limited understanding of natural history and complications*					✓			✓	✓		✓			✓	✓				✓	
Complications* of malaria should be treated with traditional remedies				✓				✓					✓		✓				✓	
Fear of adverse events including death from use of SP†																✓				
Behaviours that may reduce timely access to conventional treatment																				
Western medicines used as second resort	✓			✓		✓			✓					✓						
Health care facility used as second resort				✓		✓	✓			✓					✓		✓		✓	✓
Treatment for febrile convulsions not sought for 1-3 days																				
Medications stopped when symptoms cease					✓	✓									✓					
Hospital treatment sought after cause is relieved by traditional methods									✓						✓					
Treatment plan must be approved by fathers/husbands											✓									
Conventional treatment failure leads to switch to herbals/traditional treatment					✓															
Use of traditional/herbal remedies	✓		✓	✓	✓	✓	✓	✓	✓	✓		✓	✓	✓	✓		✓		✓	✓
Pragmatic obstacles to accessing conventional treatment																				
Distance from facility/practitioner				✓						✓					✓					✓
Costs			✓	✓			✓					✓	✓		✓			✓		

*convulsions, anemia, splenomegaly
†Sulphadoxine-pyrimethamine
